# Unusual molecular architecture of a human gut microbiota β-mannanase reveals a new CBM family

**DOI:** 10.1007/s00018-026-06241-x

**Published:** 2026-05-14

**Authors:** Natalia Łoś, Ieva Lelėnaitė, William G. T. Willats, Nicolas Terrapon, Ana Lorena Morales-García, Hamish C. L. Yau, Elisabeth C. Lowe, David N. Bolam

**Affiliations:** 1 Biosciences Institute, Medical School, Newcastle University, Newcastle upon Tyne, NE2 4HH UK; 2 School of Natural and Environmental Sciences, Newcastle University, Newcastle Upon Tyne, NE1 7RU UK; 3 Architecture et Fonction des Macromolécules Biologiques, UMR 7257, CNRS AMU; USC1408 INRAE, Marseille, 13288 France; 4 Procter & Gamble, Newcastle Innovation Centre, Newcastle upon Tyne, NE12 9TS UK

**Keywords:** Carbohydrate-binding module, β-mannanase, Bacteroidota, Glycoside hydrolase family 26, Microbiota

## Abstract

β-mannans are plant structural and storage polysaccharides prevalent in the human diet. Their degradation in the gastrointestinal tract is mediated by the human gut microbiota (HGM) through expression of a plethora of carbohydrate-active enzymes (CAZymes), although our understanding of the details of mannan breakdown is lacking. In this study, a prominent HGM member, *Bacteroides cellulosilyticus (Bc)*, was found to be exceptionally efficient at utilising β-mannans, mediated by the expression of a single polysaccharide utilisation locus (PUL). Amongst the predicted surface CAZymes encoded in the PUL, we identified a family 26 glycoside hydrolase of an unusual molecular architecture. *Bc*WH2_GH26 contains a putative carbohydrate-binding module (CBM) directly intercalated into its catalytic domain, unlike classical CBMs which are located at the N- or C-termini of the catalytic domain. Phylogenetic and functional analyses of this internal CBM, and a homologue from another mannan user *Bacteroides uniformis*, revealed a narrow specificity for β-mannans and support their classification as a novel CBM family, CBM112. To investigate the functional basis for the unusual enzyme architecture, the effect of the CBM on the catalytic activity of the enzyme was assessed. No significant differences in the kinetic parameters were found between the full-length and CBM deletion constructs against both soluble and insoluble mannans. The potential role of the internal CBM in enzyme function is discussed in the context of the likely localisation of the *Bc*WH2_GH26 in the outer membrane utilisome encoded by the *Bc* mannan PUL.

**Supplementary Information:**

The online version contains supplementary material available at 10.1007/s00018-026-06241-x.

## Introduction

Dietary fibre breakdown by the human gut microbiota plays a key role in host health through multiple mechanisms, including the production of beneficial metabolites like short chain fatty acids. Understanding the mechanisms by which fibre is broken down by the microbiota is thus key to our ability to modulate microbiota structure and composition for the benefit of health. β-mannans are a major source of dietary fibre as they are prominent components of many common foods, including legumes and seeds. In addition they are widely used in the food industry as additives such as thickeners and stabilisers [1]. Despite this prevalence in the diet, to date, only limited number of studies focused on characterisation of β-mannan utilisation systems expressed by human gut microbes, such as *Bacteroides ovatus* [2] and *Roseburia intestinalis* [3].

Structurally, β-mannan in its simplest form consists of a β1,4-linked mannose backbone (homomannan) which can either be further substituted with galactose side chains (galactomannan), consist of both mannose and glucose residues in the backbone (glucomannan), or contain both types of modifications (galactoglucomannan). In addition, glucomannans are often variably acetylated [1]. The structural complexity of the polymer was found to vary between plant species and may depend on the plant’s developmental stage [4].

Glycoside hydrolases (GHs), the largest class of CAZymes, catalyse hydrolysis of glycosidic bonds within complex carbohydrates, and are to date grouped into nearly 200 families based on sequence similarities [5]. GHs active on the β-mannan backbone belong to families 5, 26 and 113 [5]. GH26 is a well characterised family consisting primarily of endo-acting mannanases and exo-mannosidases. Many of the characterised bacterial GH26s, such as CtLIC26A (PDB: 2BV9), *B. ovatus* Man26A (PDB: 4ZXO), and *Bacillus subtilis* Man26 (PDB: 2WHK), consist of a single catalytic domain. In contrast, fungal GH26 mannanases often contain one or more carbohydrate-binding modules (CBMs) [6], often from family 35. These are located at the N- or C-terminus of the catalytic domain, in common with most CBMs [7, 8].

To expand our understanding of mannan breakdown by the gut microbiota, we investigated the ability of a number of prominent human gut Bacteroidota species to utilise mannans. Two *Bacteroides spp.*,* B. cellulosilyticus* and *B. uniformis* were particularly efficient mannan users and inspection of their predicted mannan-active surface CAZymes revealed GH26 enzymes with an unusual molecular architecture in that both enzymes contain a homologous domain of unknown function directly intercalated within their catalytic domains. Characterisation of this isolated domain revealed it to be a new family of mannan-binding CBMs, hereafter named CBM112. While the functional rationale for the unusual location of the CBM within the GH26 is not clear, it may be an adaptation to the predicted localisation of the enzyme within the surface mannan utilisomes of these species.

## Materials and methods

### Sources of carbohydrates

Mannooligosaccharides, cellooligosaccharides, carob galactomannan (low-viscosity), ivory nut mannan, konjac glucomannan (low-viscosity), wheat arabinoxylan, xyloglucan, barley and oat β-glucans were purchased from MegaZyme (Bray, Ireland). Guar gum galactomannan and hydroxyethyl cellulose were purchased from SigmaAldrich (MA, USA). Avicel was obtained from FMC Corporation (PA, USA). Insoluble mannan was obtained by washing 100 mg of ivory nut β-mannan in 1 ml deionised water five times and discarding the supernatant containing the soluble fraction of the polysaccharide. The pellet was then dried overnight at 37 °C.

### Growth array setup

Supplemented minimal medium (MM) was prepared at 2x concentration as per Table S1 and S2 with no carbohydrates added. β-mannan polysaccharides were prepared at 10 mg/ml and then mixed with the medium in 1:1 ratio, resulting in 1x MM and 5 mg/ml final carbohydrate concentration.

All growth arrays were conducted under anaerobic conditions, at 37 °C. Nineteen different Bacteroidota species (Table S3.) were initially grown overnight (16–20 h) in 5 ml of brain-heart infusion (BHI) broth (Sigma-Aldrich, MA, USA) with 1.2 µg/ml hematin and 0.2 mM histidine added.

Next, carbohydrate growth assays were conducted in triplicate in 96-wells plates. Each well contained 180 µl of 1x MM with 5 mg/ml selected carbohydrate, with an exception of three wells which did not contain any carbohydrate and served as no-carbohydrate negative controls. All the wells were then inoculated with 20 µl aliquot of the overnight culture. For the corresponding no-bacterium negative controls, 20 µl of BHI was added instead. Growth was monitored using Biotek Epoch Microplate Spectrophotometer (Agilent, CA, USA) at 15 min intervals over 48 h.

No growth of any of the bacteria was observed in the no-carbohydrate wells. The mean of OD_600_ values obtained for the no-bacterium negative controls was subtracted from the growth values of corresponding strains. The obtained blanked growth values were then plotted as growth curves using GraphPad Prism 10.6.1. (MA, USA). Lastly, the maximum OD_600_ values reached during growth were used to create a heatmap.

### Phylogenetic analysis

To build the phylogenetic tree of the CBM112 family, 82 gene sequences were obtained from the CAZy database (cazy.org) [5]. The CBM domains were identified within each gene, extracted, and aligned with MAFFT using E-INS strategy. Next, BMGE (BLOSUM62 matrix) was used to select regions within the obtained multiple sequence alignment that were most suited for phylogenetic inference. The phylogenetic tree was inferred using IQ-tree webserver. Le-Gascuel (LG) was used as the substitution matrix, with the state frequency optimised by the Maximum-Likelihood (ML). Branch support was calculated using the ultrafast analysis of 1,000 bootstrap alignments.

### Cloning

*B. cellulosilyticus *WH2 GH26 (locus tag BcellWH2_02025) full-length construct without the predicted signal peptide, referred to in the text either as *Bc*WH2_GH26 or *Bc*WH2_GH26 FL (SigP 6.0; amino acids 21–558), *Bu*_CBM (CBM only from *B. uniformis* ATCC 8492 GH26, BACUNI_00373; amino acids 240–354), and *Ruminococcus champanellensis* GH26 (RUM_21270; amino acids 32–616) were amplified by PCR from genomic DNA using KOD DNA Polymerase (Novagen) and primers presented in Table S4. The constructs were then cloned into pET-21a vector using NheI/XhoI, such that the recombinant protein encoded a C-terminal 6xHis-tag. To confirm correct insertion, the constructs were sequenced using T7 primers (Eurofins Genomics). To design the *Bc*WH2_CBM (amino acids 239–355) and the truncated *Bc*WH2_GH26_ΔCBM constructs (amino acids 21–238, 356–558), the linker sequence was first identified using AlphaFold 2 [9] full-length protein model prediction. A cut site was chosen to leave a long enough linker sequence on both constructs to minimise the risk of model misprediction and maximise the likelihood of correct protein folding. The newly designed construct sequences were then input into AlphaFold 3 [10] to predict their structures. Models of highest prediction confidence scores were chosen for cloning and expression. The chosen constructs were synthesised and inserted into a pET28a vector by Twist Bioscience with an N-terminal 6xHis-tag. The modularity of each construct is represented in Figure S1, and the full sequences are present in Table S5.

### Site-directed mutagenesis

The *Bc*WH2_CBM mutant constructs were synthesised and inserted into a pET-28a(+) vector by Twist Bioscience. In each case, a single residue (W257, W301, Y310, H339) was substituted to alanine. The rest of the CBM sequence remained unchanged. The obtained constructs contained N-terminal and C-terminal 6xHis-tag. Full sequences are present in Table S5.

### Expression and purification of recombinant proteins

All the constructs were expressed using Tuner (DE3) competent *E. coli* cells. Cells containing the recombinant plasmid were grown in 1 L Luria-Bertani (LB) broth (containing either 50 µg/ml kanamycin or 100 µg/ml ampicillin) at 37 °C with shaking at 180 rpm to mid-exponential phase (OD_600_ ~ 0.6). The recombinant protein production was induced by adding 0.2 mM final isopropyl β-d-1-thiogalactopyranoside. After incubating at 16 °C with 120 rpm agitation for 16 h, the cells were harvested, and recombinant proteins were purified using immobilized metal ion affinity chromatography (IMAC). Cell-free extract was passed through a column containing a 5 ml bed volume of cobalt-charged Chelating Sepharose Fast Flow resin (Cytiva) and washed with 20 ml of 20 mM Tris buffer pH 8.0 with 150 mM NaCl, followed by 15 ml of 10 mM imidazole in Tris buffer. The purified His-tagged proteins were eluted using 15 ml 100 mM imidazole in Tris buffer. The proteins were then dialysed into 20 mM Tris buffer pH 8.0 with 150 mM NaCl and spin-concentrated using Vivaspin™ 10,000 MW concentrator columns (Cytiva, MA, USA) and the purity of the obtained proteins was assessed using SDS-PAGE gels stained with Coomassie blue (Abcam, Cambridge, UK).

### Enzyme assays

All the enzymatic reactions were carried out at 37 °C. To investigate the product profile of the *Bc*WH2_GH26 constructs, 0.5 µM final concentration of enzyme was incubated for various time points with 5 mg/ml mannan polysaccharides in 50 mM potassium phosphate buffer, pH 7.5. The reactions were stopped by boiling for 10 min and the products analysed using high-performance anion-exchange chromatography with pulsed amperometric detection (HPAEC-PAD; Thermo ICS-6000) using Dionex CarboPac PA300-4 μm column. Detection was facilitated by a gold working electrode and a PdH reference electrode, using a standard Carbo Quad waveform. Buffer A consisted of 100 mM NaOH, Buffer B was composed of 100 mM NaOH and 0.5 M sodium acetate, and Buffer D was a 500 mM NaOH solution. The following method was used: 0–55 min of linear gradient of 50% Buffer A; 55–60 min isocratic elution with 100% Buffer B; 60–65 min isocratic elution with 100% Buffer D; and lastly 65–75 min of linear gradient of 50% Buffer A.

To determine the specific activity and *k*_cat_/*K*_M_ values of the enzymes on soluble substrates, reducing sugar assays were carried out using 3,5-dinitrosalicylic acid (DNSA), as previously described [11]. All reactions were carried out at 37 °C with constant 850 rpm agitation. Agitation was used to ensure adequate mixing of the reactions due to the viscosity of the mannans at the highest concentrations used. Each reaction had a final volume of 400 µl and contained 2.5, 3, 3.5, 4, 4.5 mg/ml of either carob galactomannan (CGM) or konjac glucomannan (KGM), and 50 mM potassium phosphate buffer, pH 7.5. The final enzyme concentration was 10 nM. At 5, 10, 15, 20 and 30 min timepoints, 60 µl aliquot was removed and the reactions were stopped by addition of DNSA reagent in a 1:1 volume ratio. The amount of reducing sugar released was quantified by measuring sample’s absorbance at 540 nm and using a standard curve of mannose. The *k*_cat_/*K*_M_ values were calculated from the initial slopes of the reactions as previously described [11], as the substrate concentrations used were significantly below the *K*_M_.

For the insoluble substrate, 3 mg/ml final concentration of insoluble ivory nut mannan was used with 1 µM final enzyme concentration in 50 mM potassium phosphate buffer, pH 7.5. The reactions were incubated at 37 °C with constant agitation (1500 rpm for determining specific activity or 100 rpm for 48 h assays) and timepoints were taken at 30, 60, 120, 180, 240, 300 min, then every hour between 23 and 29 h. One final timepoint was taken after 48 h incubation. The reactions were stopped by adding DNSA reagent in a 1:1 volume ratio to the sample, as previously described [11]. The concentration of reducing sugars was determined by measuring the *A*_540_ and comparing against a mannose standard curve. The data was then analysed using GraphPad Prism 10.6.1. (MA, USA).

### Affinity gels

Affinity gels were used for initial screening of the WT CBMs as well as initial analysis of the effects of the point mutations. Native polyacrylamide gels were made with 0.1% (w/v) final ligand concentration and 5 µg of each protein was loaded. Negative control gels did not contain ligand. Bovine serum albumin (BSA) (5 µg loaded) served as a negative control to exclude non-specific binding. Proteins were then visualised using Coomassie blue.

### Isothermal titration calorimetry

Isothermal titration calorimetry (ITC) using MicroCal PEAQ-ITC (Malvern Panalytical, Malvern, UK) was used to further investigate the specificity and affinity of the novel CBMs. Ligands were made up at either 5 mg/ml for polysaccharides or 5 mM for oligosaccharides in dialysis buffer (20 mM Tris, pH 8.0 with 150 mM NaCl). They were then injected in 18 × 2 µl aliquots into the sample cell containing 100 µM protein sample in the dialysis buffer to minimise heats of mixing. The titration was carried out at 25 °C. The fitting of the isotherm to a single set of sites binding model was carried out using MicroCal PEAQ-ITC Analysis Software v1.41. The molar concentration of the available binding sites in the polysaccharide substrates was determined by fixing the value of N to 1. This assumption was deemed valid from the structural, mutagenesis and oligosaccharide binding data showing only a single binding site on the CBMs, in addition to close to sigmodal binding curves that allowed confident determination of stoichiometry from the fitted data. This method has been used previously to determine the molar concentration of available binding sites for specific carbohydrate binding proteins on their polymeric ligands [12, 13].

### Insoluble polysaccharide binding assay

Insoluble polysaccharide binding was analysed using a pull-down assay as previously described [14], with some modifications. Briefly, 10 µg of purified CBM was incubated in 250 µl DI water, containing 4 mg insoluble ivory nut mannan (see ‘sources of carbohydrates’ above) or insoluble cellulose (Avicel), with 50 µg BSA to block non-specific binding. The negative control did not contain any ligand. The reactions were incubated for 30 min at 25°C with 900 rpm agitation. The samples were then centrifuged at 10,000 g for 15 min. Supernatant was collected and pellet was washed 5x in 20 mM Tris, pH 8.0 with 150 mM NaCl. After the final wash, 20 µl SDS loading buffer was added to the pellet. For the supernatant and negative control, 15 µl sample of each was taken and mixed with 5 µl SDS loading buffer. After boiling for 10 minutes, 12 µl of each sample was loaded on an SDS-PAGE gel. Proteins were visualised using Coomassie blue.

### Preparation of carbohydrate microarrays and microarray probing

Carbohydrate microarrays were prepared as previously described [15]. Briefly, glycan standards were dissolved in deionised water to a final concentration of 2 mg/mL and placed on a rotary shaker at 4 °C for 16 h to ensure complete solubilisation. Glycan solutions were added to the wells of 384-well microtiter plate and diluted 1:1 (v/v) with printing buffer (47% glycerol, 52.9% deionised water, 0.06% Triton X-100 and 0.04% Proclin™ 200) and printed onto nitrocellulose membrane using a non-contact microarray robot (‘Marathon Argus’, Arrayjet, Roslin, UK).

Probing of printed glycoarrays was performed according to Johnsen et al. (2015) [16] with slight modifications. Briefly, microarrays were blocked for 1 h at room temperature in 1x PBS supplemented with 5% (w/v) semi-skimmed milk powder (MP/PBS). Following incubation, arrays were probed for 2 h with the following probes: *Bc*WH2_CBM (His-tag); characterised β-mannan-binding CBM27A (His-tag, NZYtech) [17]; anti-(1,4)-β-mannan BS-400-4 (mouse, BioSupplies); anti-heteromannan LM21 (rat) and anti-galactomannan CCRC-M75 (mouse, CarboSource) monoclonal antibodies. BS-400-4 was used at 40 µg/ml, CCRC-M75 and LM21 (hybridoma supernatant) at 1:50 dilution, *Bc*WH2_CBM at 10 µM (140 µg/ml), and CBM27A at 10 µg/ml. Microarrays were washed thoroughly in clean 1x PBS and then incubated for 2 h with alkaline phosphatase conjugated secondary antibodies (anti-His tag in the case of CBMs and anti-rat or anti-mouse in the case of mAbs) diluted 1:1000 in MP/PBS. mAb and binding was then detected by 5-bromo-4-chloro-3’-indolyphosphate p-toluidine (BCIP) salt and nitro-blue tetrazolium (NBT) chloride. Membranes were rinsed in fresh water and allowed to dry overnight. Microarray probing was performed in triplicate for each probe and control. Developed arrays were scanned at 2400 dpi and converted to negative JPEG files. Binding intensities of probes to individual spots was quantified using microarray analysis software (Array-Pro Analyzer Version 6.3). A value of 100 was assigned to the highest mean spot signal intensity recorded and all other values were normalised accordingly [18].

## Results

### Identification of β-mannan PULs in *Bacteroides cellulosilyticus* and *Bacteroides uniformis*

**Fig. 1:**
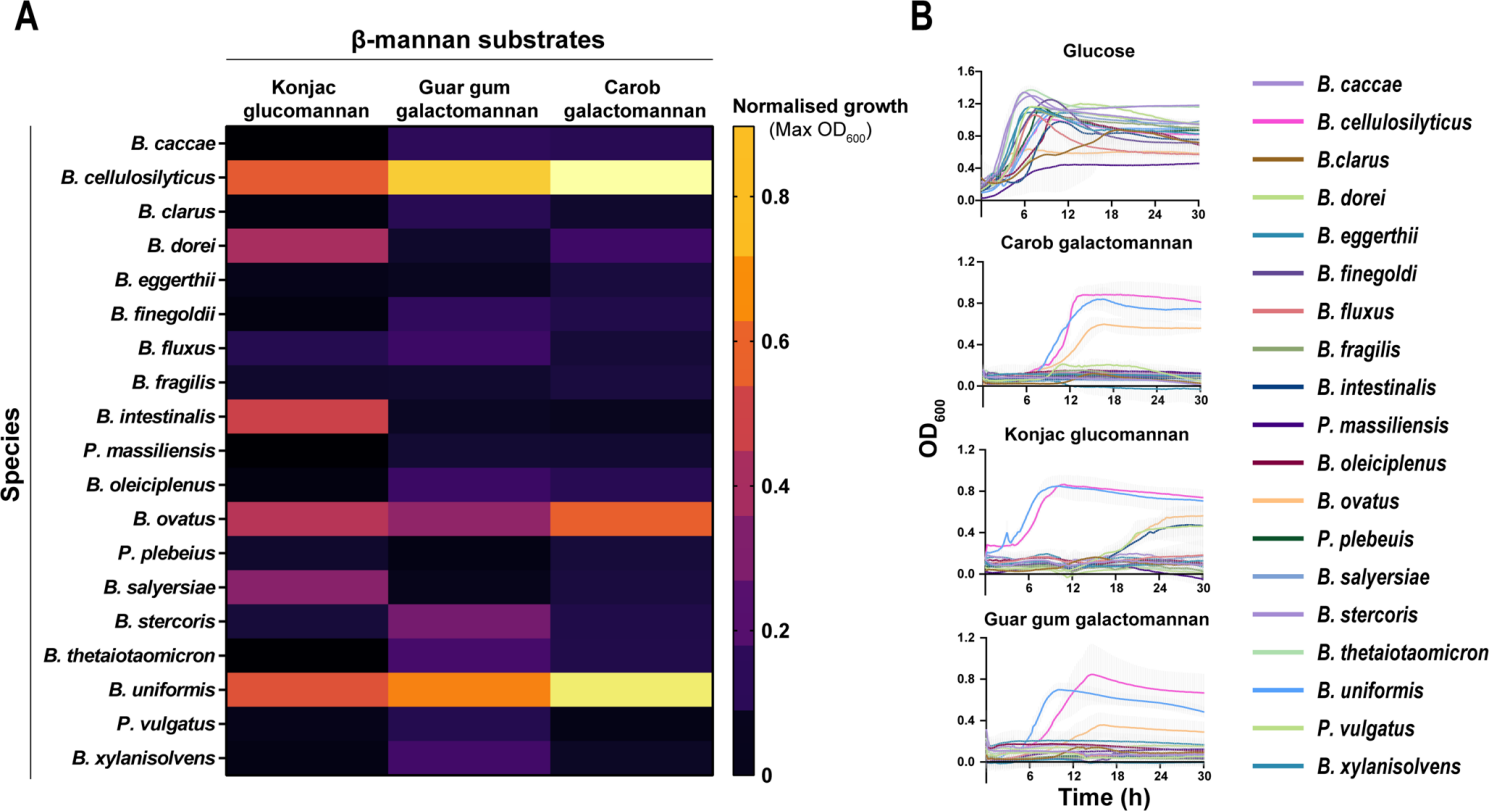
Growth of different gut Bacteroidota species on β-mannans. **A**) Heatmap showing maximum OD_600_ values reached by 19 human gut *Bacteroides* and *Phocaeicola* species when grown on 5 mg/ml β-mannan substrates – konjac glucomannan, guar gum galactomannan, and carob galactomannan. Bacteria were grown in 96-well plate (200 µl well volume) over a course of 48 h. The OD_600_ values of negative controls were subtracted before plotting. **B**) Corresponding growth curves used to plot the heatmap.

In pursuit of characterising novel β-mannan degradation mechanisms, we cultivated a total of 19 different human gut *Bacteroides* and *Phocaeicola* species using three types of β-mannan (carob galactomannan [CGM], konjac glucomannan [KGM], and guar gum galactomannan [GGM]) as a sole carbon source, Fig. 1. Interestingly, despite both genera being known to be versatile glycan utilisers [19], we found that the majority of the tested species were unable to use all of the β-mannans for growth, suggesting a lack of β-mannan-specific enzymatic machinery, Fig. 1A. Only three species were able to grow on all types of β-mannan tested – *B. cellulosilyticus*,* B. uniformis*,* and B. ovatus*, Fig. 1B. The former two utilised β-mannan most efficiently, as their growth was characterised by shortest lag phase, fastest exponential phase, and highest max OD reached, Fig. 1B.

**Fig. 2:**
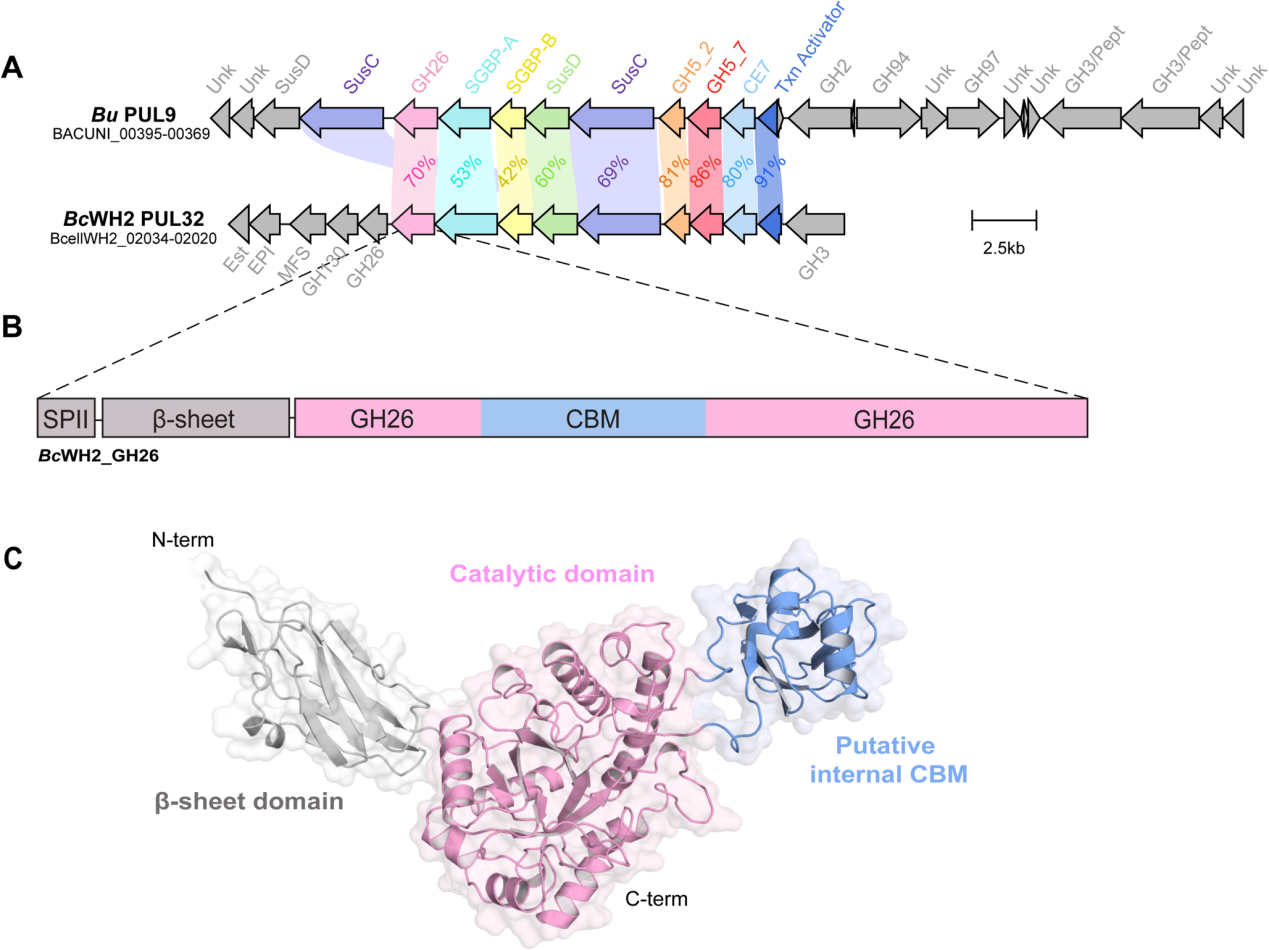
*B. cellulosilyticus* and *B. uniformis* β-mannan PULs and molecular architecture of *Bc*WH2_GH26. **A**) Map of *Bc*WH2 PUL32 and *Bu* PUL9, both containing GH26 enzymes with internal domains predicted to be CBMs. The composition of *Bc*WH32 PUL32 was determined experimentally, with all the genes shown to be upregulated during the bacterium’s growth on β-mannans [20]. The composition of the syntenic *Bu* PUL9 was predicted using PULDB [22]. To indicate similarities between the two PULs, genes sharing a minimum of 30% amino acid sequence identity have been aligned and highlighted in different colours, with the % identity shown. The remaining non-homologous genes are shown in grey. Created and visualised using Clinker [23]. **B**) Schematic representation of the molecular architecture of *Bc*WH2_GH26. The enzyme consists of a type II signal peptide (SPII, in grey), an N-terminal β-sheet domain of unknown function, hypothesised to be a spacer domain involved in positioning the enzyme in the surface utilisome (in grey), a catalytic GH26 domain (in pink), and a domain shown here to be an internal CBM (in blue). **C**) A model of the *Bc*WH2_GH26 structure, generated using AlphaFold2 [9]. Signal peptide was removed, and each domain was coloured as per schematic above, i.e. β-sheet domain in grey, catalytic domain in pink, and CBM in blue.

To investigate the mechanisms underlying these two species ability to efficiently degrade mannans, we examined their genomes for potential mannan targeting enzymes. With *B. cellulosilyticus* WH2 a previous study has shown that PUL32 (spanning locus tags BcellWH2_02034 − 02020) was amongst the most highly upregulated during growth on β-mannans [20]. This large and complex PUL is predicted to contain multiple mannan active CAZymes, including two GH26s (Table S6). Bioinformatic analysis then allowed us to identify a syntenic PUL in *B. uniformis* – Bu PUL9 (locus tags BACUNI_00395-BACUNI_00369). The two PULs share multiple homologous genes (highlighted in Fig. 2), however, there are also key differences in their compositions. Notably, *Bc*WH2 PUL32 lacks multiple predicted exo-glycosidases present in the *Bu* PUL9 (GH2, GH97, GH3/Peptidases), and the two PULs contain different phosphorylase families (GH130_1 in *Bc*WH2 PUL32 and GH94 in *Bu* PUL9). Despite these differences, the two PULs do share a prominent feature – they both contain homologous GH26 enzymes of an unusual molecular architecture, Fig. 2. An enzyme with similar architecture is missing from *B. ovatus* β-mannan PUL and all other *Bacteroides* strains tested in this study. Furthermore, the two enzymes – *Bc*WH2_GH26 (locus tag: BcellWH2_02025) and *Bu*GH26 (BACUNI_00373) – are predicted to be localised to the outer membrane (based on their type II signal peptide), suggesting a potential key role in the initial surface β-mannan breakdown. However, their most interesting feature is a previously uncharacterised domain of unknown function (DUF), inserted directly into the peptide sequence of their catalytic module, Fig. 2. While the overall catalytic activity of *Bu*GH26 has previously been described [21], no functional characterisation of its internal domain was carried out. Both DUF domains have a β-sandwich-like fold with surface aromatics forming a potential binding cleft, an architecture common to many CBMs. Based on this we suspected the internal DUF of these enzymes could be a novel family of CBMs.

### Internal DUFs of *Bc*WH2_GH26 and *Bu*_GH26 are highly specific β-mannan binding CBMs

**Fig. 3:**
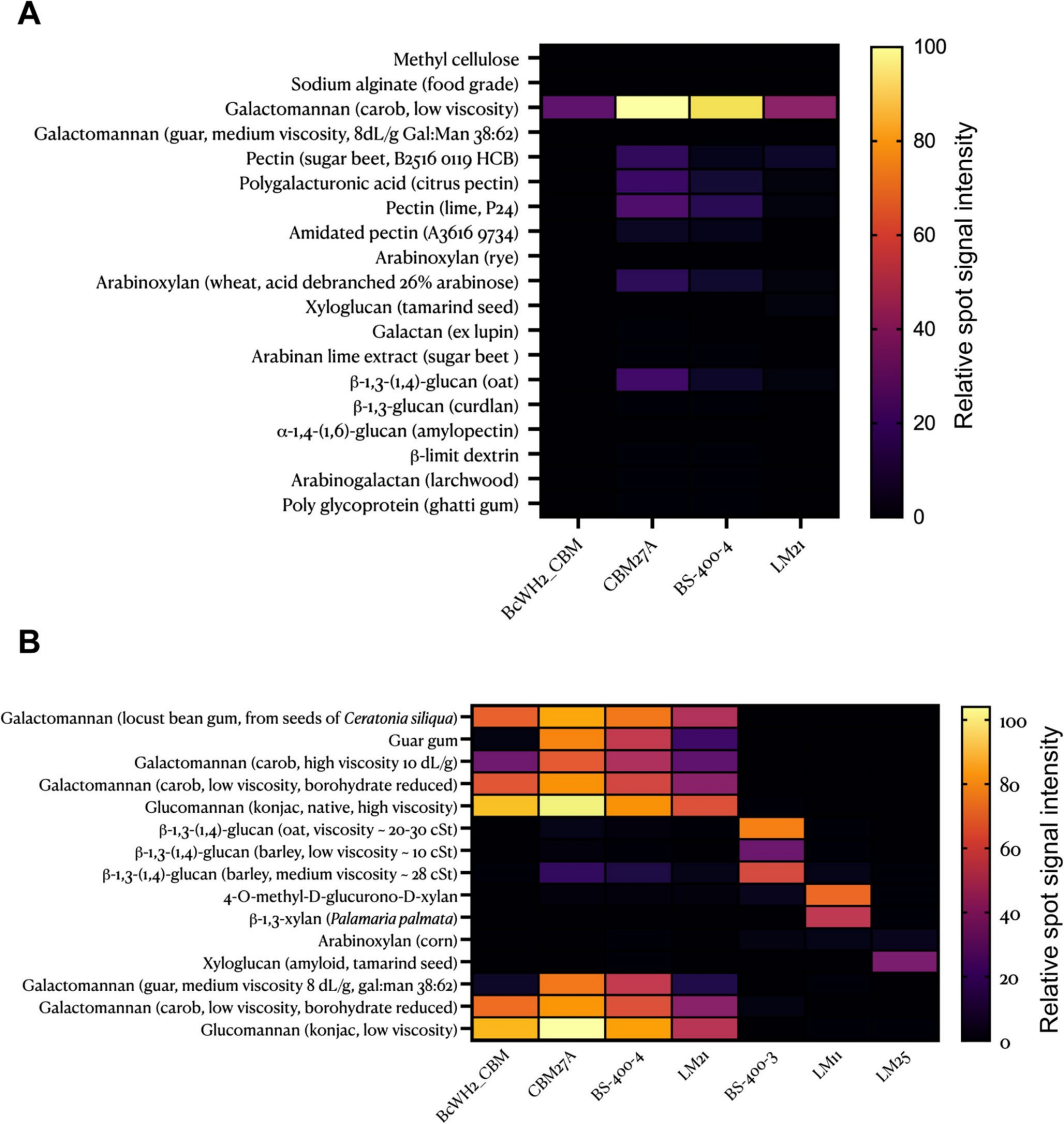
Binding specificity screening of *Bc*WH2_CBM using carbohydrate microarrays. Carbohydrate microarrays were used to obtain heatmaps showing results of broad-scope screening of *Bc*WH2_CBM to diverse polysaccharide standards (**A**) and more mannan-focused binding specificity screening (**B**). The polysaccharide standards are listed on the left. The mannan binding probes CBM27 and mAbs BS-400-4 and LM21 were used as positive controls, and mAbs BS-400-3, LM11 and LM25 were used as negative controls. Data are relative mean spot signals obtained from three independent printing and probing experiments and on each array, carbohydrate standards were printed as 3 technical replicates. The highest mean signal in the dataset was set to 100, and all other values were normalised accordingly.

Using carbohydrate microarrays, the putative internal CBM of *Bc*WH2_GH26 was initially screened against a broad range of polysaccharides selected as representatives of major glycan classes, and its binding specificity was compared to CBM27A (a known mannan binder) and well-characterised mannan-specific monoclonal antibodies (mAbs), BS-400-4 and LM21, 3A. This initial screen showed *Bc*WH2_CBM binding to CGM but not GGM. *Bc*WH2_CBM was more selective than CBM27A, which in addition to binding CGM, also showed signs of weak binding to several other glycans such as pectin and arabinoxylan, Fig. 3A.

Next, the binding specificity of *Bc*WH2_CBM was investigated in more detail using a range of β-linked hemicelluloses, Fig. 3B. *Bc*WH2_CBM bound to all CGM preps tested but failed to bind to GGM, in contrast to CBM27A which bound to all types of galactomannan. *Bc*WH2_CBM also showed binding to KGM, but did not bind any glucans or xylans, further highlighting its narrow β-mannan specificity.

The results of the carbohydrate microarray screening were further confirmed by running native affinity gels. Both *Bc*WH2_CBM and its close homologue from *B. uniformis* GH26, *Bu*_CBM (~ 70% identity), bound CGM and KGM, but failed to bind to barley mixed-linkage β-glucan and wheat arabinoxylan, Figure S2.

### Assessing the affinity of *Bc*WH2_CBM and *Bu*_CBM

**Fig. 4:**
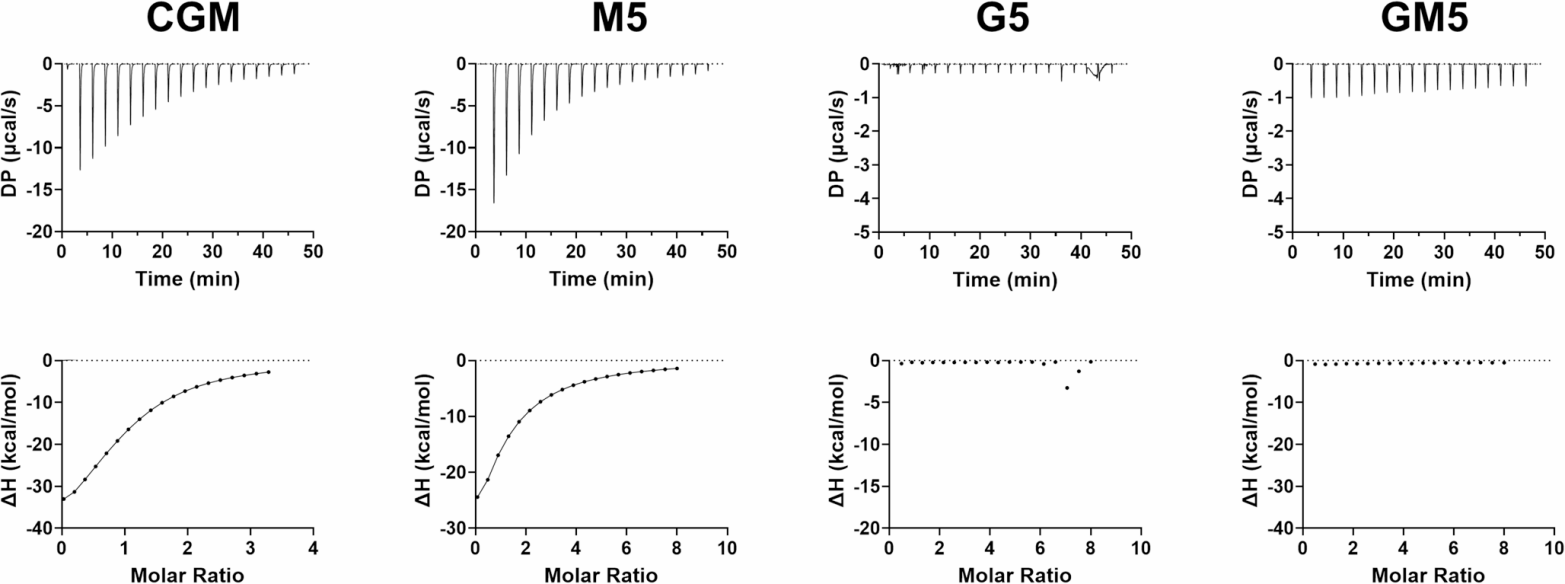
Example ITC of *Bc*WH2_CBM interactions with selected ligands. Glycans used are carob galactomannan (CGM), mannopentaose (M5), cellopentaose (G5), and di-alpha-D-galactosyl-mannopentaose (GM5). Titrations were conducted at 25 °C in 20 mM Tris buffer, pH 8.0, containing 150 mM NaCl. Upper parts of each panel are raw binding heats, lower parts are integrated data fit to single set of sites model if binding observed.

To further examine the specificity and affinity of these novel CBMs, isothermal titration calorimetry (ITC) was performed using a range of β-linked polysaccharides. *Bc*WH2_CBM and its homologue, *Bu*_CBM, were found to bind to both CGM and KGM, with comparable affinity, Table 1. Selected example ITC titrations are shown in Fig. 4, with further ITC titrations shown in Figures S3 and S4 and full binding parameters set shown in Table S7.

**Table 1:** Affinity of *Bc*WH2_CBM and *Bu*_CBM binding to oligo- and polysaccharides determined by ITC^a^

Construct	Ligand	K_D_ (µM) ± SD	*N* ± SD
*Bc*WH2_CBM^b^	Carob galactomannan	67 (± 6)	1^d^
	Konjac glucomannan	52 (± 7)	1^d^
	Mannohexaose (M6)	132 (± 34)	1.3 (± 0.3)
	Mannopentaose (M5)	235 (± 3)	1 ± (0.3)
	Mannotetraose (M4)	445 (± 21)	1.7 (± 0.1)
	Mannotriose (M3)	NB^e^	-
	Hydroxyethylcellulose (HEC)	NB^e^	-
	Barley β-glucan	NB^e^	-
	Xyloglucan	NB^e^	-
	Cellopentaose	NB^e^	-
	6^3^,6^4^-di- α-D-galactosyl-mannopentaose	NB^e^	-
*Bu*_CBM^c^	Carob galactomannan	38 (± 6)	1^d^
	Konjac glucomannan	47 (± 21)	1^d^
	Mannohexaose (M6)	43 (± 2)	0.8 (± 0.1)
	Mannopentaose(M5)	82 (± 6)	1.3 (± 0.4)
	Mannotetraose (M4)	335 (± 26)	0.7 (± 0.4)
	Mannotriose (M3)	NB^e^	-
	Hydroxyethylcellulose (HEC)	NB^e^	-
	Xyloglucan	NB^e^	-
	Cellopentaose	NB^e^	-
	6^3^,6^4^-di- α-D-galactosyl-mannopentaose	NB^e^	-

^a^ Parameters determined by ITC. Standard deviation calculated for the means of at least triplicate titrations

^b^ *Bc*WH2_GH26 isolated CBM

^c^ *Bu*_GH26 isolated CBM

^d^ N was fixed to 1 for polysaccharides (see Methods)

^e^ No binding detected

ITC with defined oligosaccharides revealed the minimal chain length required for binding was four mannoses, as the modules failed to bind to mannotriose. The chain length of the ligand did substantially influence the modules binding affinity, with *Bc*WH2_CBM affinity for M5 (*K*_D_ = 235 µM) decreasing two-fold compared to M6 (*K*_D_ = 132 µM) and further two-fold for M4 (*K*_D_ = 438 µM), suggesting the full binding site spanned at least 6 mannoses, although the number of binding sites on the CBM (N) for all manno-oligosaccharides was close to 1 for both CBMs, Table 1. Interestingly despite binding glucomannan, neither CBM bound any of the β-glucan homopolymers tested or cellopentaose. Furthermore, both while both CBMs could bind galactomannan, they were unable to accommodate di-galactosyl side chains on neighbouring mannose residues, as shown by lack of binding to 6^3^,6^4^-di- α-D-galactosyl-mannopentaose, despite binding M5. *Bc*WH2_CBM preference for less-decorated polysaccharides was also confirmed via native affinity gels, where a higher degree of retardation was observed for carob galactomannan (4:1 Man: Gal) compared to guar gum galactomannan (Man: Gal 2:1), Figure S2.

### *Bc*WH2_CBM is able to bind insoluble β-mannan

**Fig. 5:**
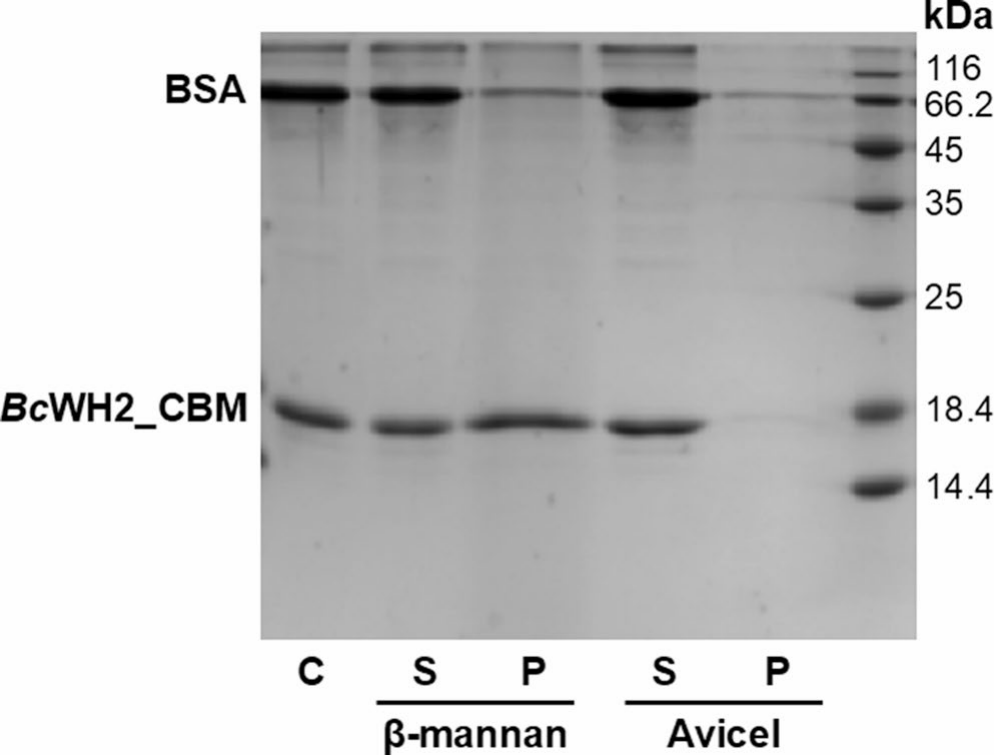
Binding *Bc*WH2_CBM to insoluble β-mannan. After incubating *Bc*WH2_CBM with insoluble ivory nut mannan (β-mannan) or cellulose (Avicel), the samples were centrifuged to separate supernatant (S) and pellet (P) fractions. The control (C) sample did not contain any polysaccharide. Bovine serum albumin (BSA) was included in each assay to minimise non-specific binding and as a non-interacting control. All samples were subsequently analysed by SDS-PAGE. Molecular weight standards are shown on the right.

One of the classical roles of CBMs is thought to be to potentiate the activity of the cognate catalytic domain against insoluble substrates [24]. To investigate the ability of *Bc*WH2_CBM to bind insoluble polysaccharides we carried out pulldown assays with the insoluble fraction of ivory nut β-mannan (INM) and microcrystalline cellulose (Avicel). Bovine serum albumin (BSA) was added to each reaction to minimise the effects of non-specific binding and as a non-interacting control. The results revealed that *Bc*WH2_CBM is able to bind insoluble β-mannan but not cellulose, as evidenced by a band on the gel in the pellet fraction of mannan, but not Avicel, Fig. 5. Furthermore, the faint BSA band in both pellet fractions was of similar intensity, indicating that the binding to mannan was specific to the CBM. However, a CBM band was observed in the supernatant fraction of mannan showing that not all of the CBM was bound to the mannan in this experiment, possibly due to the mannan being saturated with CBM. Overall these data are supportive of *Bc*WH2_CBM being able to bind insoluble mannan, but not cellulose, further confirming the CBMs tight specificity for mannan.

### *Bc*WH2_CBM and *Bu*_CBM are founding members of a novel CBM family

**Fig. 6:**
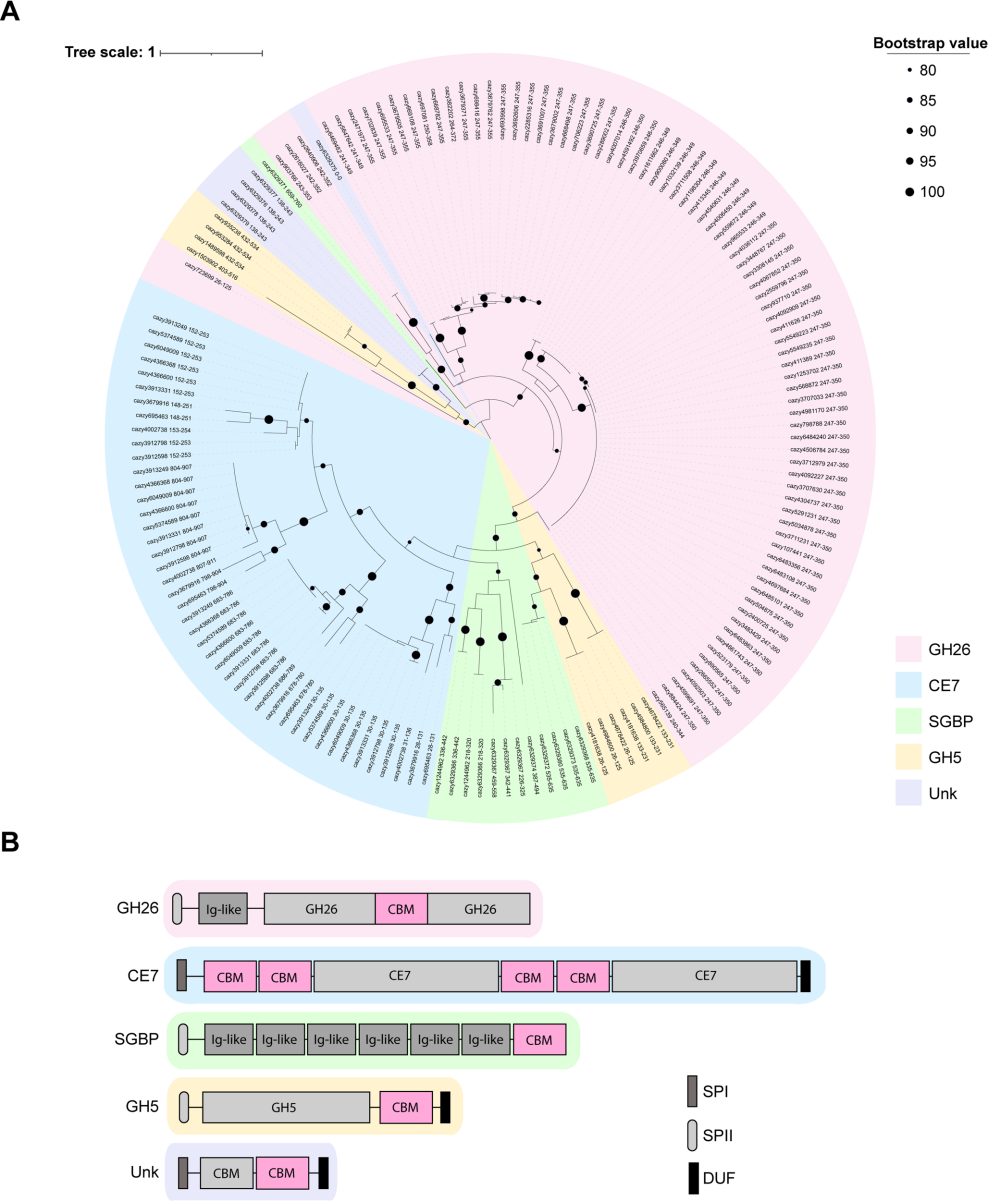
Phylogenetic analysis of the CBM112 family. **A**) Phylogenetic tree of all members of the CBM112 family. Sequences obtained from the CAZy database [5] and annotated with their CAZy database entry names. Each protein class associated with the CBM112 is indicated by a different colour, as follows: GH26 in pink, CE7 in blue, putative surface glycan-binding proteins (SGBP) in green (function hypothesized based on their sequence and structural similarity to previously characterised SGBPs), GH5 in yellow, and proteins of unknown function in purple. Nodes with bootstrap values greater than 80 are indicated by black circles of increasing size, as per legend. **B**) Schematic of domain architecture of representatives of each protein class associated with CBM112. CBM112 is highlighted in pink, the remaining domains are depicted in shades of grey/black. Type of signal peptide (SPI/SPII) and predicted protein functions have been annotated (GH26, CE7, GH5, CBM). Ig-like β-sheet domains present in putative SGBPs and GH26s are depicted as grey boxes. Additionally, CE7, GH5, and proteins annotated here as Unknown (Unk), all contain a C-terminal domain of around 30–40 amino acids of unknown function. These C-terminal modules are annotated here as domains of unknown function (DUF) and depicted as black rectangles.

Upon confirming *Bc*WH2_CBM and *Bu*_CBM to be novel β-mannan binding CBMs, a phylogenetic analysis was performed. This allowed for the establishment of a novel CBM family, CBM112. Searching CAZy and PULDB databases [5, 22], we found 82 proteins to contain the CBM112 modules, Fig. 6A. The majority of these were Bacteroidota proteins, found in the human and mammalian gut and oral cavity. CBM112 modules can be appended to various protein classes. Most often, they are intercalated into the catalytic domain of GH26 modules of *Bacteroidales* spp (*Bacteroides*, *Prevotella*, and *Duncaniella*) analogously to *Bc*WH2_GH26. In rare cases, such as in GH26 from *Prevotella sp.* AGR2160 (RefSeq. WP_081657628.1) the catalytic domain remains uninterrupted and is instead preceded by the CBM, indicating the CBM is not absolutely required to be located internal to the catalytic domain for functionality. CBM112 is also found associated with esterases, such as CE7 from *Segatella copri* (WP_317576045.1). In this case, two repeats of the CBM domain are followed by a CE7 module and this structure then repeats, Fig. 6B. Less commonly, CBM112 can be appended at the C-term to a GH5. Unlike the CBM112 containing GH26s, these GH5s do not possess an extra Ig-like N-term domain. Interestingly, CBM112 are also found in putative surface glycan-binding proteins (SGBPs), based on the protein molecular architecture (multiple Ig-fold domains followed by the CBM at the C-term) and position of the gene within the PUL downstream of the *susD*. Lastly, in rare cases, members of this family can be found as discrete entities alongside another domain of a predicted structure suggesting a CBM of an unknown family. These double CBM proteins contain type I signal peptide, type (e.g., GTC17253_17770), suggesting a different role to the other family members. Overall, this analysis suggests that CBM112’s function may not be solely limited to the classical model of the CBM assisting the catalytic activity of enzyme. Aiming to investigate this further, we then looked at a broader genomic context of these CBMs.

### CBM112 is most often found within predicted β-mannan PULs

To examine the broader context in which CBM112s function, we carried out genomic analysis. This revealed that 80–94% of the CBM112 family members are found within a CAZyme/PUL context. Out of the 82 proteins containing CBM112 module, 64 are located within classical PULs, and 2 are associated within CAZyme clusters. Furthermore, 11 out of the 16 remaining members are found within the neighbourhood of CAZyme genes such as GH26 and GH130, however not in a close enough proximity to be considered a cluster. Interestingly, four of the CBM112s not found in the context of a PUL are the aforementioned discrete CBMs, not attached to any other protein class.

**Fig. 7:**
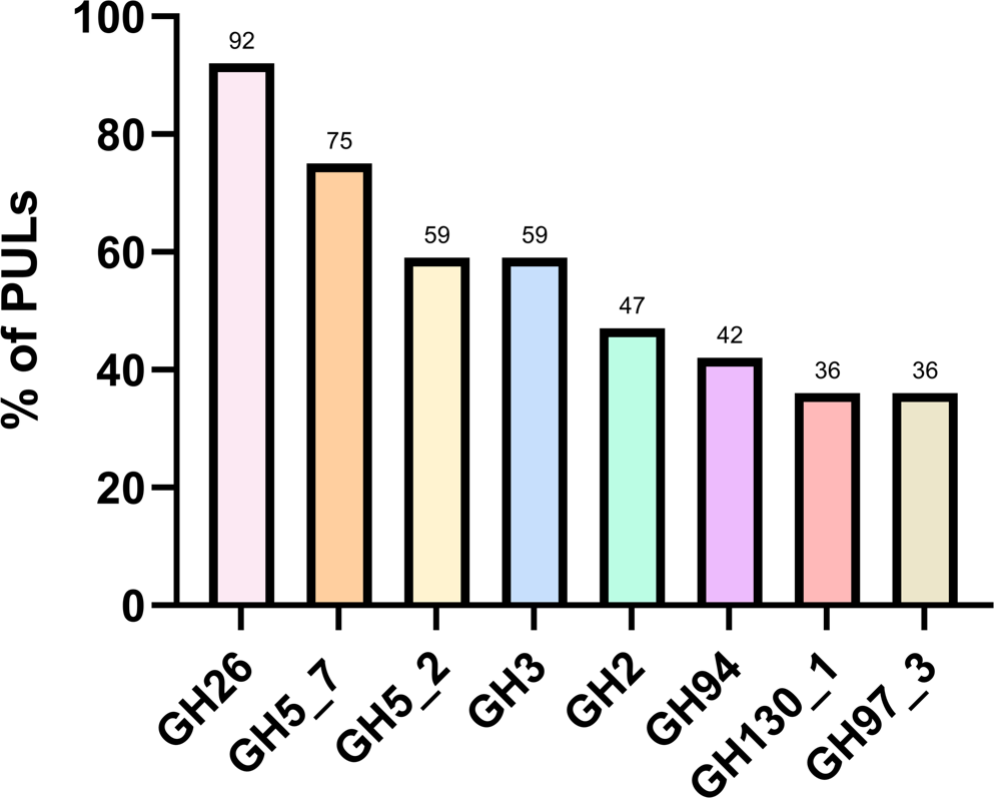
Co-occurrence of different GH families in PULs containing CBM112. Depicted here as percentage of occurrence of a given GH family in all PULs in PUL-DB containing CBM112 [22]. Data show that GH26 is a family most often associated with CBM112 PULs, as they can be found in 91% of these PULs. Other enzymes most commonly found within these PULs belong to families GH5_7 (found in 75% of CBM112 PULs), GH5_2 (59%), GH3 (59%), GH2 (47%), GH94 (42%), and GH130_1 and GH97_3 (36% each). Based on the activities previously observed in these families, these data suggest that CBM112s are most often found in putative β-mannan or β-glucomannan targeting PULs.

Focusing on the classical PULs, we found that although their exact composition varies, some patterns can be discerned. The GH family most often observed to co-occur alongside CBM112 are GH26, which were found in 91% of PULs containing the CBM112, Fig. 7. This was followed by GH5_7 present in 78% of CBM112 PULs, and GH5_2 and GH3, each found in 59% of PULs. Other co-occurring enzymes belong to families GH2 (47%), GH94 (42%), GH130_1 (36%) and GH97_3 (36%). GH26 and GH5_7 families predominantly consist of β-mannanases, meanwhile GH5_2 and GH3 are often involved in β-glucan degradation [5]. Based on these previously observed family activities, our data suggest that CBM112s are most often found in β-mannan or β-glucomannan targeting PULs. The latter is further supported by the presence of GH130_1 enzyme in over a third of CBM112-containing PULs, as this GH130 subfamily contains solely mannosylglucose phosphorylases [5].

Interestingly, our analysis did not reveal GH36 to be associated with the CBM112 PULs, even though this family of α-galactosidases is often involved in debranching galactomannans [25]. Therefore, we speculate that either the specificity of the CBM112 PULs may lean more towards linear mannan/glucomannan utilisation, as opposed to galactomannan; or these PULs might instead utilise GH97 to remove α-galactose side chains, although this family has not previously been shown to act on β-mannan. Alternatively, the PUL enzymes might cooperate with a GH36 located outside the PUL. This may be the case for *B. cellulosilyticus*, where an orphan GH36 is upregulated during the growth on β-mannan at the same time as PUL32 containing CBM112 [20].

### Determining key ligand binding residues in *Bc*WH2_CBM112

**Fig. 8:**
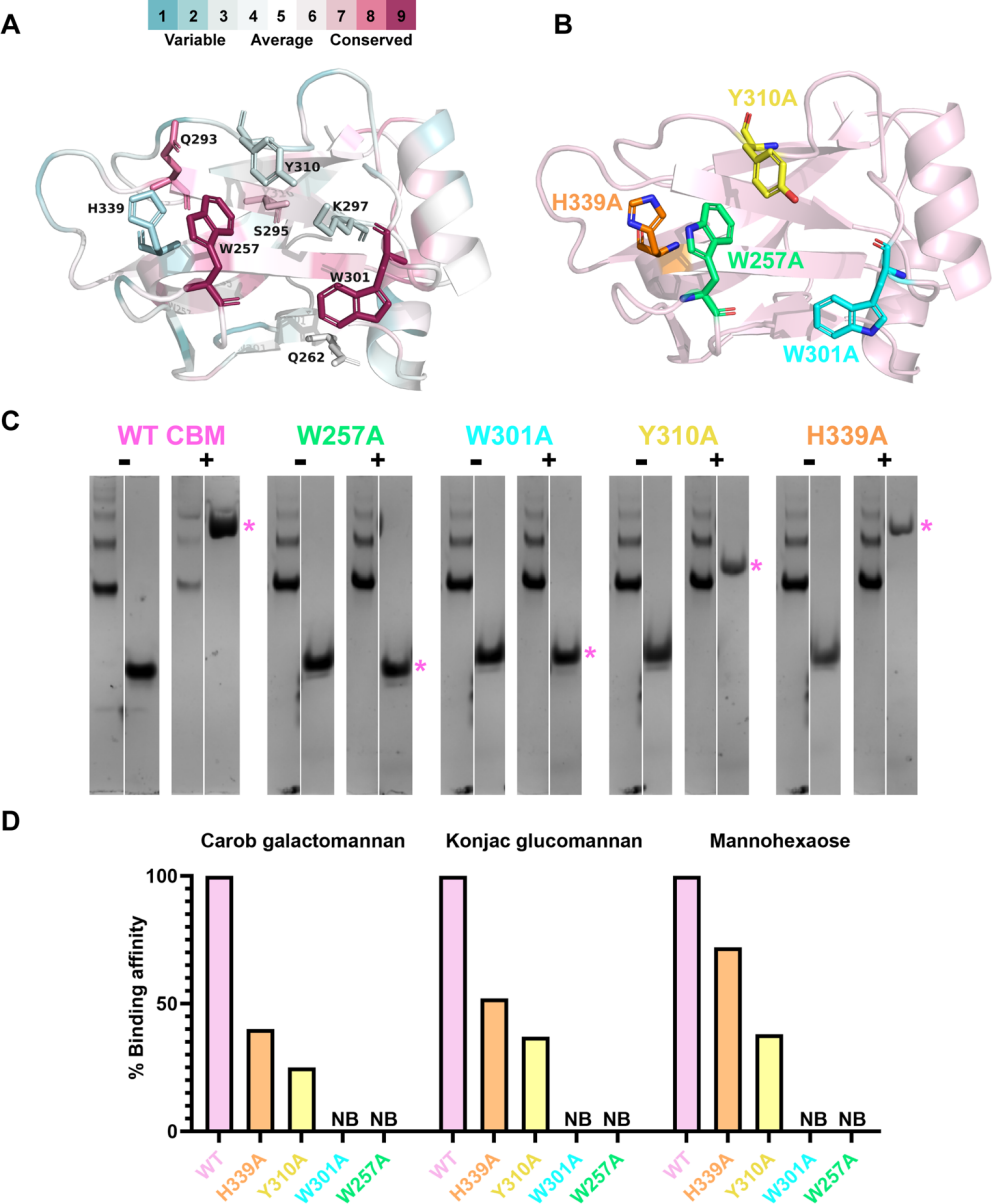
Identification of *Bc*WH2_CBM ligand binding residues. **A**) Conservation of proposed binding site residues created using ConSurf [26]. Multiple-sequence alignment of the CBM112 family was used as a template. Conservation grades scale is shown. **B**) Highlighted residues within the proposed *Bc*WH2_CBM112 binding site, selected for mutagenesis **C**) Native gel containing 0.1% (w/v) konjac glucomannan (+) or no ligand (-) showing affinity of wild-type *Bc*WH2_CBM112 (WT) and its corresponding mutants to this ligand. BSA was used as a negative control and is shown on the left of each gel. The positioning of the CBM in each ligand-containing gel is indicated by a pink asterisk. **D**) *K*_D_ values of WT *Bc*WH2_CBM112 and its corresponding mutants for three ligands, shown here as percentage of the WT *K*_D_ value. Full dataset showing exact *K*_D_ values is presented in Table S8. NB indicated no binding was detected.

The putative ligand binding site of the *Bc*WH2_CBM112 was predicted based on the AlphaFold2 prediction of the protein structure. The degree of conservation of residues within the binding cleft varies, however, the two surface exposed tryptophan residues (W257 and W301) are conserved across the whole CBM112 family, suggesting their key role in ligand binding, Fig. 8A. To experimentally confirm this, we made alanine mutants of four *Bc*WH2_CBM residues located within the putative binding site - W257, W301, Y310 and H339, Fig. 8B.

Affinity gel electrophoresis with glucomannan revealed that W257A and W301A mutants displayed no binding to the polysaccharide, meanwhile Y310A retained limited binding capacity and the H339A mutant bound similarly to WT on the gels, Fig. 8C and S5.

To obtain more specificity and affinity data, the binding of the mutants to a range of oligo- and polysaccharides was assessed via ITC and compared against the wild-type protein, Fig. 8D and Table S8. Once again, no binding was observed for W257A and W301A mutants on any of the ligands tested. The H339A mutant was able to retain almost half of the WT CBM’s binding affinity to the polysaccharides tested, however, interestingly, the mutation had less of an effect when protein was assayed against mannohexaose. This may be explained by the location of the H339 residue within the binding site and the differences in length between the ligands tested. Meanwhile, Y310A mutation decreased the ability of the CBM to bind its ligands, including mannohexaose, almost four-fold, Fig. 8D.

Overall, these data confirm W257 and W301 to be key binding residues in BcWH2_CBM112. Without the stacking interactions provided by them, the CBM completely fails to bind to its ligands. Y310 was found to also be important in ligand binding, however, to a lower degree than the two tryptophan residues. Due to its orientation within the binding site, we suspect that Y310 does not participate in stacking interactions, and instead, is involved in hydrogen bonding with the sugar, Fig. 8B. Similarly, H339 is most likely involved in H-bonding with ligand, albeit, to a lesser extent.

### The effect of the CBM on the enzyme’s activity vs. soluble and insoluble mannans

To investigate the effect of the CBM112 on the activity and product profile of *Bc*WH2_GH26, a truncated construct of the enzyme was created, lacking the internal CBM (*Bc*WH2_GH26_ΔCBM). To ensure the stability of this construct, we performed nanoDSF scan of both proteins and did not observe significant differences in their melting temperatures, Figure S6.

The full-length enzyme (*Bc*WH2_GH26 FL) and *Bc*WH2_GH26_ΔCBM were incubated overnight with either CGM, KGM or M6, and the reaction products were analysed via HPAEC-PAD. No differences in the oligosaccharide products released by the two constructs were seen, Figure S7. Both enzyme variants released a mixture of oligosaccharides of various degree of polymerisation (DP), with the main products ranging in length from DP2 to DP6. The retention time of the oligosaccharides obtained from CGM and KGM hydrolysis differed compared to the linear standards, suggesting that they were either GlcMan heterooligosaccharides (for KGM) or branched oligosaccharides (for CGM), however, their exact identity has not been elucidated. Overall, the obtained data indicate an endo-acting mode of action of *Bc*WH2_GH26, as previously observed in enzyme’s homologs such as *Buni*_GH26 [21] and GH26B from *Bacteroides ovatus* [25].

Next, the catalytic efficiency (*k*_cat_/*K*_M_) was determined for both the full-length protein and its truncated version against soluble substrates – CGM and KGM. Overall, both constructs were ~ 1.5-2-fold more active against KGM compared to CGM, Table 2. No significant differences were seen when comparing the two constructs on either CGM or KGM (paired t test p value > 0.05), further evidencing that the CBM does not increase the efficiency of the enzyme against soluble substrates. The linear regressions used to estimate *k*_cat_/*K*_M_ are shown in Figure S8.

**Table 2:** Catalytic efficiency of *Bc*WH2_GH26 FL and *Bc*WH2_GH26_ΔCBM against soluble mannans

Substrate	Construct	k_cat_/K_M_ g/litre^− 1^ s^− 1^
Carob galactomannan	*Bc*WH2_GH26 FL	46 (± 5) ^a^
	*Bc*WH2_GH26_ΔCBM	48 (± 6) ^a^
Konjac glucomannan	*Bc*WH2_GH26 FL	77 (± 6) ^a^
	*Bc*WH2_GH26_ΔCBM	110 (± 9) ^a^

^a^ Data are averages and std deviation of triplicate measurements

To test the effect of the CBM on insoluble substrate, specific activity of the two *Bc*WH2_GH26 constructs was determined using a reducing sugar assay with 3 mg/ml final concentration of the insoluble fraction of the ivory nut β-mannan (INM). The results were then compared against those obtained for equivalent concentration of soluble substrates (CGM and KGM), revealing, as would be expected, higher hydrolysis rates against soluble substrates compared to insoluble INM, Table 3. The rates of the two constructs were near identical at the initial stages of the reactions, Figure S9, resulting in only marginal differences in the calculated specific activity against all three substrates, including INM, Table 3. After incubating insoluble mannan assays for 48 h at lower rotation speed (100 rpm) to avoid protein denaturation, we failed to observe any differences in the amount of reducing sugar released by each of the enzymes, Figure S10A. However, we did observe that both constructs were able to fully solubilise INM, resulting in clarification of the solution. Nonetheless, upon assaying *Ruminococcus champanellensis* GH26 (*Rc*GH26; RUM_21270) containing an N-term CBM, we found that this enzyme was also able to degrade INM to the same extent, Figure S10B, therefore confirming that ability to solubilise INM is not unique to *Bc*WH2_GH26.

**Table 3:** Specific activity of two *Bc*WH2_GH26 enzyme constructs on soluble and insoluble mannans

	Specific activity s^− 1^	
Substrate^a^	BcWH2_GH26 FL^b^	BcWH2_GH26_ΔCBM
Insoluble β-mannan	0.3 ± 0.006	0.4 ± 0.05
Carob galactomannan	171 ± 22	133 ± 22
Konjac glucomannan	241 ± 33	293 ± 65

^a^0.3% w/v β-mannans. ^b^ Slope values were normalised to the enzyme concentration used

### Proposed ligand binding model for CBM112

**Fig. 9:**
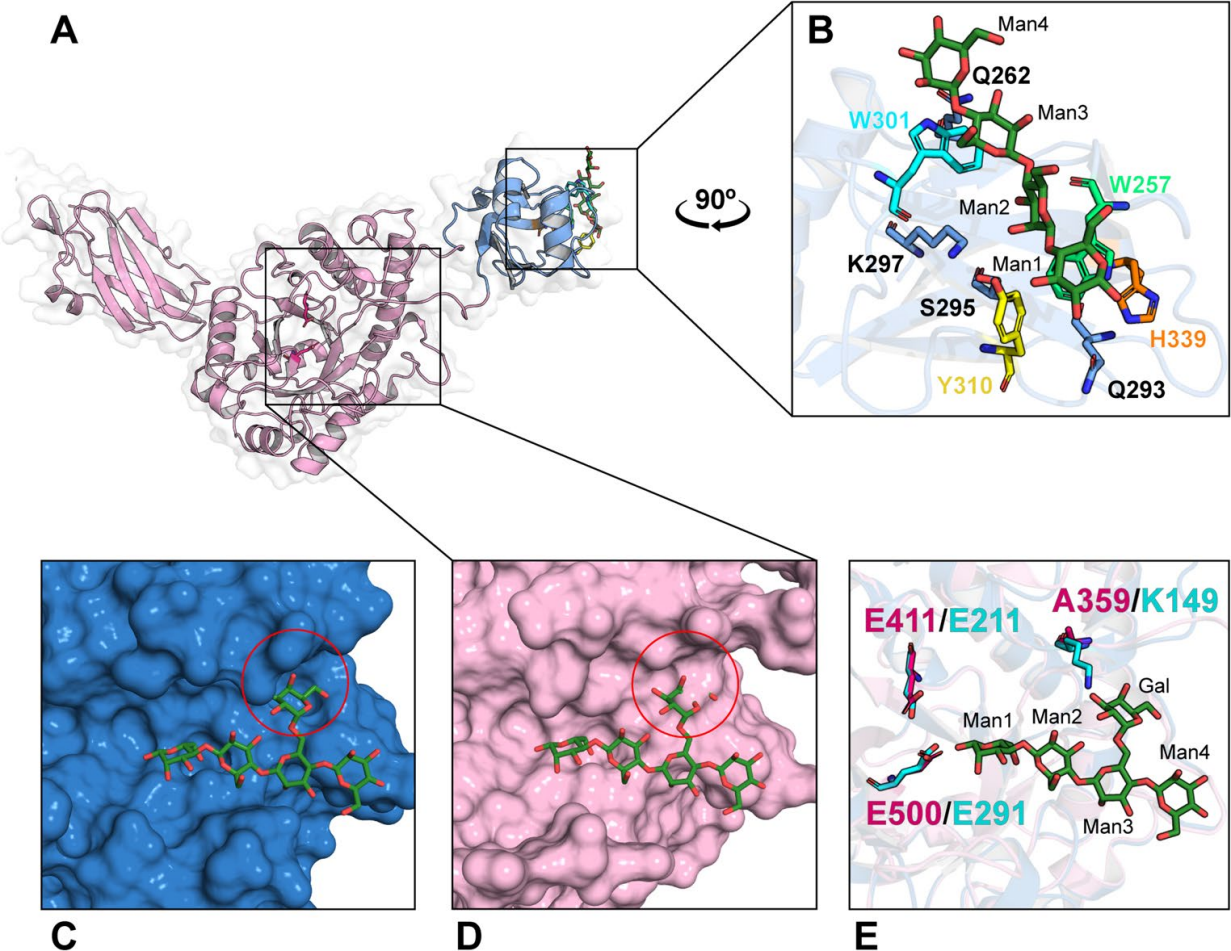
Model of *Bc*WH2_GH26 catalytic domain and CBM112 bound to mannooligosaccharides. **A**) AlphaFold2 model of a full-length enzyme with a docking prediction of its CBM (in blue) bound to linear mannotetraose (M4) created using Chai-1 Discovery software [27]. **B**) Close-up of the CBM112 binding site with M4 (in green) modelled in. Binding residues highlighted. **C**) Crystal structure of *Bo*Man26B (PDB: 6HF4) active site with a decorated galactomanno-oligosaccharide (M4 with single Gal side chain at Man3; G1M4) bound. Red circle highlights structural pocket al.lowing accommodation of galactose side chain on Man3. **D**) Model of a *Bc*WH2_GH26 active site with a G1M4 from PDB 6HF4 overlaid into active site. Red circle indicates the Y201 residue causing steric clash with the galactose side chain. **E**) Superimposition of *Bc*WH2_GH26 and *Bo*Man26B active sites with G1M4 bound. *Bo*Man26B catalytic residues (E211 and E291) are shown in cyan. Also shown is K149 (cyan) in *Bo*GH26 that forms a hydrogen bond with the galactose side chain. Highlighted in pink are putative catalytic residues (E411 and E500) of *Bc*WH2_GH26 and alanine residue (A359) in the analogous position to *Bo* K149, preventing hydrogen bond formation with the galactose side chain of Man3.

As crystallisation trials to obtain a ligand complex were unsuccessful, we used in silico tools to generate a model of *Bc*WH2_GH26 CBM112 bound to mannotetraose, Fig. 9A. The location of the CBM binding site and the positioning of the residues in relation to the ligand are consistent with the data obtained from analysis of the CBM point mutants. In common with most CBM binding sites, tryptophan residues (W257 and W301 in *Bc*WH2_GH26 CBM112) play a key role in ligand recognition by forming CH–π stacking interactions with the mannose rings of Man1 and Man3 (labelled from the reducing to non-reducing end), Fig. 9B. The spatial arrangement of the OH-6 group of Man1 relative to W257 suggests that the addition of a galactosyl side chain at this position would introduce a steric clash, thereby impeding binding and, consequently, providing rationale for the inability of the CBM to accommodate heavily-galactosylated ligands.

Furthermore, the positioning of Y310 supports its involvement in hydrogen bonding with the sugar, most likely via interaction between its phenolic hydroxyl group and OH-2 of Man2. Such structural arrangement could impact ligand specificity at this subsite, i.e., an alternative C2 hydroxyl configuration, such as equatorial OH-2 of glucose, could cause a steric clash at this subsite and thus preclude binding.

Other residues in *Bc*WH2_CBM112 binding cleft capable of hydrogen bond formation, which could influence ligand specificity include Q262, Q293, S295, K297, and H339 (Fig. 9B), the last of which has been experimentally shown to be involved in ligand binding, Fig. 8, most likely via interaction with OH-1 of Man1.

Besides the two tryptophans, Q293 is the amongst the most conserved residues within the binding cleft, suggesting it might play a key role in determining ligand specificity, Fig. 8A. It most likely interacts with Man1 and its location at the periphery of the binding cleft provides structural rationale for DP4 being the minimum chain length required for binding, despite the apparent suitability of mannotriose to span the two tryptophan residues (W257 and W301), Fig. 9B.

To investigate the relative positioning of the CBM binding site in the context of the full length enzyme the model of the *Bc*WH2_GH26 catalytic domain was overlaid with the crystal structure of *B. ovatus Bo*Man26B (PDB: 6HF4) consisting of a singular domain (52% amino acid sequence identity shared with a catalytic domain of *Bc*WH2_GH26), bound to a decorated galactomanno-oligosaccharide (M4 with single Gal side chain at Man3; G1M4), Fig. 9A. This revealed that the CBM’s binding site appears to face in the opposite direction and orientation to the catalytic domain’s binding cleft, suggesting that the CBM does not act as a direct extension of the enzyme’s active site i.e. appears unlikely to bind to the same mannan chain as the enzyme.

### Accommodation of decorated mannans by *Bc*WH2_GH26 catalytic domain

Overlay of the active sites of the two *Bacteroides* GH26 enzymes (*Bc*WH2_GH26 and *Bo*Man26B) also revealed some interesting differences in their ability to accommodate decorated mannans, Fig. 9C-E. Although the catalytic residues (E411 and E500 for *Bc*WH2_GH26) are conserved as expected within the same family, the two enzymes differ in their predicted − 4 subsites. The tyrosine residue (Y201) in *Bc*WH2_GH26 clashes with the Gal decoration on the M3 of the overlaid G1M4, as indicated by a red circle in Fig. 9D. Steric clashes with potential O6 Gal decorations might also occur at the Man1 due to the presence of Y524. Based on the overlay, the galactose side chains could therefore be accommodated at Man2 and Man4, but they cannot be localised on two neighbouring mannose residues.

Furthermore, in the case of *Bo*Man26B, K149 forms hydrogen bonds with a galactosyl-side chain of Man3 [25], whereas, in *Bc*WH2_GH26, this residue is substituted with alanine suggesting no direct interaction between the *Bc*WH2_GH26 and galactose at this site.

Overall, these structural features preventing binding of galactosyl-side chains at multiple subsites in *Bc*WH2_GH26, confer the enzyme unable to act upon highly decorated regions of galactomannans. This is thus consistent with the previously observed inability of its CBM to bind highly galactosylated mannooligosaccharide ligands.

However, all of this analysis is based on modelled sugars in the CBM and sugar from an overlay with the catalytic domain and thus caution must be taken with the conclusions drawn.

## Discussion

In this study, we functionally characterised two founding members of a novel CBM family – CBM112. CBM112s tested in this study are type B CBMs, able to bind both soluble and insoluble mannans [28]. Interestingly, both CBM112s exhibited very narrow range of specificity, binding solely to β-mannan, and failing to interact with other β-linked polysaccharides tested, Table 1. This stands in contrast with many other characterised β-mannan binding CBMs, which display higher degree of ligand promiscuity. For instance, members of family CBM29, are able to bind β-mannan, but also β-linked glucans and cellulose, due to very few hydrogen bonds being formed between their binding site residues and the C2 hydroxyl of the ligand, Figure S11 [29]. In contrast, *Bc*WH2_CBM112 binding site contains multiple residues capable of forming hydrogen bonds (H339, Y310, Q262, Q293, K297, S295), and their importance in ligand binding has been confirmed via affinity studies of targeted mutants (Y310 and H339), as per Fig. 8. We speculate, based on the Chai1 binding model, Fig. 9, that the positioning of Y310 could potentially limit the ligand specificity of the CBM, preventing binding of glucose at this subsite, with β1,4-linked mannosyl-saccharides as the only conformationally compatible ligands. This is further supported by ITC data, which show that *Bc*WH2_CBM112 is unable to bind G5 despite binding M5, highlighting the likely involvement of C2 hydroxyl configuration in ligand specificity. Nevertheless, *Bc*WH2_CBM112 and *Bu*_CBM112 were both able to bind glucomannan, a polymer consisting of both glucose and mannose residues. These observations indicate that the CBMs either exhibit specificity for backbone regions composed exclusively of mannose residues, or alternatively, they demonstrate a capacity to accommodate glucose residues at certain subsites, provided that mannose occupies the critical positions essential for binding. This represents a distinct recognition mechanism compared to the previously characterized glucomannan-binding CBM16 and CBM29 families, which can accommodate either hexose residue within the key binding sites, as demonstrated by their capacity to bind both cello- and manno-oligosaccharides, Figure S11 [30, 31].

Moreover, both CBM112 modules tested failed to bind 6³,6⁴-di-galactosyl-mannopentaose (G2M5), despite binding to CGM, Table 1. This observation further supports the notion that their capacity to accommodate main chain substitutions is restricted only to specific subsites within the binding cleft. For instance, based on the Chai1 model, we suspect that the positioning of W257 might preclude accommodation of galactosyl-side chain at Man1. This is not unusual and has been previously observed within the binding site of CBM27, in which the galactosyl-substitutions can only be tolerated in subsites 1, 2, 3 and 5, consequently, explaining this CBMs low affinity for G2M5, Figure S11 [17]. CBM112 displaying a similar pattern of discrimination of side chains to CBM27 agrees with the observed lower affinity for the highly galactosyl-decorated GGM compared to CGM, as shown by native affinity gels, Figure S2. This specificity for relatively unsubstituted galactomannan regions is mirrored in the BcWH2_GH26 catalytic domain. Comparison between predicted model of *Bc*WH2_GH26 and crystal structure of *Bo*GH26B, suggests that the *Bc*WH2_GH26 active site might be unable to accommodate galactosyl-side chains, due to a steric hindering caused by Tyr210, Fig. 9. Combining the experimental data with the computational structural predictions, we speculate that although *Bc*WH2_GH26 and its CBM are able to target galactomannans, they can only access less decorated regions of these storage polysaccharides. A preference for low-substituted galactomannan substrates has previously been described in other mannanases, such as *B. ovatus* GH26A enzyme (*Bo*Man26A) which is thought to act in synergy with a GH36 α-galactosidase from the same PUL to degrade galactosylated substrates [2]. In contrast to the *B. ovatus* β-mannan PUL, *Bc*PUL32 does not contain a GH36, however, a non-PUL GH36 gene was found to be highly upregulated during *B. cellulosilyticus* growth on galactomannan [20].

One of the most interesting aspects of the two CBMs tested in this study is their unusual positioning within the enzyme. Although uncommon, the existence of internal CBMs has been previously reported in several GH families, such as GH10 xylanases [32], GH13 amylases [33], and GH148 glucanases [34]. Most recently, a structural homolog of *Bc*WH2_GH26 from *B. uniformi*s has been identified, however, no functional characterisation of its internal CBM had been undertaken [21]. Despite this, the rationale for evolving such molecular architecture over the more classical terminal CBM arrangement remains poorly understood.

To investigate the CBMs biological role, enzymatic activity of the full-length *Bc*WH2_GH26 and *Bc*WH2_GH26_ΔCBM was compared. Interestingly, no significant differences were observed between the two constructs when comparing their activity on either soluble or insoluble substrates (Table 3). The lack of difference in activity against insoluble mannan indicates that *Bc*WH2_CBM112 might not play the canonical role of a CBM, which is to enhance catalytic performance by targeting the enzyme to the hard-to-access polysaccharides embedded within cell walls. This is perhaps not surprising considering the constrained positioning of the *Bc*WH2_CBM within the enzyme, which will restrict movement of the catalytic domain once the CBM is bound to mannan. This stands in contrast to terminally located CBMs which are often connected to the catalytic domains via flexible linkers, allowing independent movement of the two domains [35, 36].

Furthermore, it is worth noting that the majority of studies assessing the effect of CBMs on enzymes’ catalytic efficiency focused on cellulose-binders [24, 37, 38], with only limited data available on β-mannan-binders [7, 39] and all of them being terminal CBMs. Insoluble mannan is less crystalline than cellulose and thus less recalcitrant to enzyme degradation, as shown by our data.

Nonetheless, the ability of *Bc*WH2_CBM to bind insoluble mannan suggests its potential involvement in targeting of insoluble substrates in an alternative way. Considering the human gut environment and its short transit time compared to, for example, ruminants, the CBM might be an adaptation for efficient degradation of insoluble fibres derived from our diet. The lack of an effect on the activity of its corresponding enzyme could be attributed to the nature of the simple model substrate used, which is less complex than the heterogenous plant cell wall.

Moreover, to fully understand the function of this CBM and rationale for developing such a unusual molecular architecture, the broader context of the likely cellular location of the *Bc*WH2_GH26 enzyme needs to be considered. Based on the composition of *Bc* PUL32, *Bc*WH2_GH26, alongside either one or both SGBPs, most likely forms part of a β-mannan utilisome [40]. The location of the CBM, as well as the presence of the N-terminal β-sheet domain might therefore both be structural adaptations helping position the enzyme within utilisome complex.

The CBM internal positioning as a utilisome adaptation is further evidenced when comparing other enzymes possessing similar domain architecture. Both *B. thetaiotaomicron* SusG and *Pseudacanthotermes militaris* GH10 contain internal CBMs and are located within PULs containing SGBPs [32, 41], strongly suggesting they form complexes analogous to *B. cellulosilyticus* β-mannan utilisome. Since internal CBMs are found within various enzyme classes, they are most likely not a polysaccharide-specific adaptation. Nonetheless, they likely function to improve the catalytic efficiency of the utilisation system, providing the bacterium with a competitive advantage when growing on a specific polysaccharide.

To investigate whether all CBM112 modules function in a context of a utilisome, we performed genomic analysis of the family. We found that 61 out of 82 proteins associated with CBM112s are GH26s of a structure homologous to *Bc*WH2_GH26, i.e., that contain type II signal peptide and both the N-terminal domain and an internal CBM. The majority of these are located within PULs and therefore, most likely function within a utilisome.

This notion is further reinforced by the instances in which the CBM112 is not associated with a GH26, and instead, serves as the glycan binding domain of putative SGBPs, such as B5F25_13220 or Bacsa_1712, Fig. 6B. These SGBPs are located within PULs also containing GH26 genes. The GH26s (e.g., B5F25_13225 or Bacsa_1713) are structural homologues of *Bc*WH2_GH26, but do not contain the internal CBM. Provided that these proteins are all components of the same outer membrane utilisomes this could indicate that the role of CBM112 depends on its specific location in the utilisome rather than within the GH26 per se. It should be noted, however, that this hypothesis does not extend to all members of the CBM112 family, as the present discussion focuses specifically on the domains associated with GH26s and SGBPs localised in PULs. A subset of CBM112 is found associated with proteins predicted to be periplasmic rather than localised to the outer membrane, such as CE7s, in which case the CBM likely plays a different role.

Overall, the results of this study provide initial insight into novel β-mannan-active enzymes and their function within complex degradation systems. These insights contribute to our understanding of glycan breakdown by gut microbes, essential for the development of novel, diet-based, health-promoting interventions.

Furthermore, as β-mannanases are extensively used in various industries (e.g., oil drilling, biofuel production and paper bleaching) [42], the unusual molecular architecture of the mannanases described here may be exploited to enhance their usefulness in these and other applications. Indeed, recent studies using molecular probes identified β-mannans as prominent contributors to hard-to-remove household stains [43], highlighting the potential role of highly efficient mannanases in detergent compositions.

## Supplementary Information

Below is the link to the electronic supplementary material.

Supplementary Material 1

## Data Availability

The data supporting the findings of this study are available within the article and in its Supplementary Information.

## References

[1] Moreira LR, Filho EX (2008) An overview of mannan structure and mannan-degrading enzyme systems. Appl Microbiol Biotechnol 79(2):165–178 18385995 10.1007/s00253-008-1423-4

[2] Bågenholm V, Reddy SK, Bouraoui H, Morrill J, Kulcinskaja E, Bahr CM et al (2017) Galactomannan Catabolism Conferred by a Polysaccharide Utilization Locus of Bacteroides ovatus: ENZYME SYNERGY AND CRYSTAL STRUCTURE OF A β-MANNANASE. J Biol Chem 292(1):229–243 27872187 10.1074/jbc.M116.746438 PMC5217682

[3] La Rosa SL, Leth ML, Michalak L, Hansen ME, Pudlo NA, Glowacki R et al (2019) The human gut Firmicute Roseburia intestinalis is a primary degrader of dietary β-mannans. Nat Commun 10(1):905 30796211 10.1038/s41467-019-08812-y PMC6385246

[4] Liepman AH, Nairn CJ, Willats WG, Sørensen I, Roberts AW, Keegstra K (2007) Functional genomic analysis supports conservation of function among cellulose synthase-like a gene family members and suggests diverse roles of mannans in plants. Plant Physiol 143(4):1881–1893 17307900 10.1104/pp.106.093989 PMC1851810

[5] Drula E, Garron ML, Dogan S, Lombard V, Henrissat B, Terrapon N (2022) The carbohydrate-active enzyme database: functions and literature. Nucleic Acids Res 50(D1):D571–d7 34850161 10.1093/nar/gkab1045 PMC8728194

[6] von Freiesleben P, Moroz OV, Blagova E, Wiemann M, Spodsberg N, Agger JW et al (2019) Crystal structure and substrate interactions of an unusual fungal non-CBM carrying GH26 endo-β-mannanase from Yunnania penicillata. Sci Rep 9(1):2266 30783168 10.1038/s41598-019-38602-x PMC6381184

[7] von Freiesleben P, Spodsberg N, Stenbæk A, Stålbrand H, Krogh K, Meyer AS (2018) Boosting of enzymatic softwood saccharification by fungal GH5 and GH26 endomannanases. Biotechnol Biofuels 11:194 30026809 10.1186/s13068-018-1184-y PMC6048861

[8] Couturier M, Roussel A, Rosengren A, Leone P, Stålbrand H, Berrin JG (2013) Structural and biochemical analyses of glycoside hydrolase families 5 and 26 β-(1,4)-mannanases from Podospora anserina reveal differences upon manno-oligosaccharide catalysis. J Biol Chem 288(20):14624–14635 23558681 10.1074/jbc.M113.459438 PMC3656314

[9] Jumper J, Evans R, Pritzel A, Green T, Figurnov M, Ronneberger O et al (2021) Highly accurate protein structure prediction with AlphaFold. Nature 596(7873):583–589 34265844 10.1038/s41586-021-03819-2 PMC8371605

[10] Abramson J, Adler J, Dunger J, Evans R, Green T, Pritzel A et al (2024) Accurate structure prediction of biomolecular interactions with AlphaFold 3. Nature 630(8016):493–500 38718835 10.1038/s41586-024-07487-w PMC11168924

[11] Rogowski A, Briggs JA, Mortimer JC, Tryfona T, Terrapon N, Lowe EC et al (2015) Glycan complexity dictates microbial resource allocation in the large intestine. Nat Commun 6:7481 26112186 10.1038/ncomms8481 PMC4491172

[12] Szabo L, Jamal S, Xie H, Charnock SJ, Bolam DN, Gilbert HJ et al (2001) Structure of a family 15 carbohydrate-binding module in complex with xylopentaose. Evidence that xylan binds in an approximate 3-fold helical conformation. J Biol Chem 276(52):49061–49065 11598143 10.1074/jbc.M109558200

[13] Cartmell A, Lowe EC, Baslé A, Firbank SJ, Ndeh DA, Murray H et al (2017) How members of the human gut microbiota overcome the sulfation problem posed by glycosaminoglycans. Proc Natl Acad Sci U S A 114(27):7037–7042 28630303 10.1073/pnas.1704367114 PMC5502631

[14] Liberato MV, Campos BM, Tomazetto G, Crouch LI, Garcia W, Zeri ACM et al (2022) Unique properties of a Dictyostelium discoideum carbohydrate-binding module expand our understanding of CBM-ligand interactions. J Biol Chem 298(5):101891 35378128 10.1016/j.jbc.2022.101891 PMC9079177

[15] Vidal-Melgosa S, Pedersen HL, Schückel J, Arnal G, Dumon C, Amby DB et al (2015) A new versatile microarray-based method for high throughput screening of carbohydrate-active enzymes. J Biol Chem 290(14):9020–9036 25657012 10.1074/jbc.M114.630673 PMC4423690

[16] Johnsen HR, Striberny B, Olsen S, Vidal-Melgosa S, Fangel JU, Willats WG et al (2015) Cell wall composition profiling of parasitic giant dodder (Cuscuta reflexa) and its hosts: a priori differences and induced changes. New Phytol 207(3):805–816 25808919 10.1111/nph.13378

[17] Boraston AB, Revett TJ, Boraston CM, Nurizzo D, Davies GJ (2003) Structural and thermodynamic dissection of specific mannan recognition by a carbohydrate binding module, TmCBM27. Structure 11(6):665–675 12791255 10.1016/s0969-2126(03)00100-x

[18] Moller I, Marcus SE, Haeger A, Verhertbruggen Y, Verhoef R, Schols H et al (2008) High-throughput screening of monoclonal antibodies against plant cell wall glycans by hierarchical clustering of their carbohydrate microarray binding profiles. Glycoconj J 25(1):37–48 17629746 10.1007/s10719-007-9059-7 PMC2234451

[19] Pudlo NA, Urs K, Crawford R, Pirani A, Atherly T, Jimenez R et al (2022) Phenotypic and Genomic Diversification in Complex Carbohydrate-Degrading Human Gut Bacteria. mSystems 7(1):e00947–e00921 35166563 10.1128/msystems.00947-21 PMC8845570

[20] McNulty NP, Wu M, Erickson AR, Pan C, Erickson BK, Martens EC et al (2013) Effects of diet on resource utilization by a model human gut microbiota containing Bacteroides cellulosilyticus WH2, a symbiont with an extensive glycobiome. PLoS Biol 11(8):e1001637 23976882 10.1371/journal.pbio.1001637 PMC3747994

[21] Qu Z, Liu H, Yang J, Zheng L, Huang J, Wang Z et al (2025) Selective utilization of medicinal polysaccharides by human gut Bacteroides and Parabacteroides species. Nat Commun 16(1):638 39809740 10.1038/s41467-025-55845-7 PMC11733155

[22] Terrapon N, Lombard V, Drula É, Lapébie P, Al-Masaudi S, Gilbert HJ et al (2018) PULDB: the expanded database of Polysaccharide Utilization Loci. Nucleic Acids Res 46(D1):D677–D683 29088389 10.1093/nar/gkx1022 PMC5753385

[23] Gilchrist CLM, Chooi Y-H (2021) clinker & clustermap.js: automatic generation of gene cluster comparison figures. Bioinformatics 37(16):2473–2475 33459763 10.1093/bioinformatics/btab007

[24] Hervé C, Rogowski A, Blake AW, Marcus SE, Gilbert HJ, Knox JP (2010) Carbohydrate-binding modules promote the enzymatic deconstruction of intact plant cell walls by targeting and proximity effects. Proc Natl Acad Sci U S A 107(34):15293–15298 20696902 10.1073/pnas.1005732107 PMC2930570

[25] Bågenholm V, Wiemann M, Reddy SK, Bhattacharya A, Rosengren A, Logan DT et al (2019) A surface-exposed GH26 β-mannanase from Bacteroides ovatus: Structure, role, and phylogenetic analysis of BoMan26B. J Biol Chem 294(23):9100–9117 31000630 10.1074/jbc.RA118.007171 PMC6556568

[26] Ben Chorin A, Masrati G, Kessel A, Narunsky A, Sprinzak J, Lahav S et al (2020) ConSurf-DB: An accessible repository for the evolutionary conservation patterns of the majority of PDB proteins. Protein Sci 29(1):258–267 31702846 10.1002/pro.3779 PMC6933843

[27] team CD, Boitreaud J, Dent J, McPartlon M, Meier J, Reis V et al (2024) Chai-1: Decoding the molecular interactions of life. bioRxiv. 2024.10.10.615955

[28] Kognole AA, Payne CM (2018) Cellulose-specific Type B carbohydrate binding modules: understanding oligomeric and non-crystalline substrate recognition mechanisms. Biotechnol Biofuels 11:319 30519283 10.1186/s13068-018-1321-7 PMC6267901

[29] Charnock SJ, Bolam DN, Nurizzo D, Szabó L, McKie VA, Gilbert HJ et al (2002) Promiscuity in ligand-binding: The three-dimensional structure of a Piromyces carbohydrate-binding module, CBM29-2, in complex with cello- and mannohexaose. Proc Natl Acad Sci U S A 99(22):14077–14082 12391332 10.1073/pnas.212516199 PMC137839

[30] Bae B, Ohene-Adjei S, Kocherginskaya S, Mackie RI, Spies MA, Cann IKO et al (2008) Molecular Basis for the Selectivity and Specificity of Ligand Recognition by the Family 16 Carbohydrate-binding Modules from Thermoanaerobacterium polysaccharolyticum ManA*. J Biol Chem 283(18):12415–12425 18025086 10.1074/jbc.M706513200

[31] Freelove AC, Bolam DN, White P, Hazlewood GP, Gilbert HJ (2001) A novel carbohydrate-binding protein is a component of the plant cell wall-degrading complex of Piromyces equi. J Biol Chem 276(46):43010–43017 11560933 10.1074/jbc.M107143200

[32] Wu H, Ioannou E, Henrissat B, Montanier CY, Bozonnet S, O’Donohue MJ et al (2021) Multimodularity of a GH10 Xylanase Found in the Termite Gut Metagenome. Appl Environ Microbiol 87(3):e01714–e01720 33187992 10.1128/AEM.01714-20 PMC7848910

[33] Koropatkin NM, Smith TJ (2010) SusG: a unique cell-membrane-associated alpha-amylase from a prominent human gut symbiont targets complex starch molecules. Structure 18(2):200–215 20159465 10.1016/j.str.2009.12.010

[34] Angelov A, Pham VTT, Übelacker M, Brady S, Leis B, Pill N et al (2017) A metagenome-derived thermostable β-glucanase with an unusual module architecture which defines the new glycoside hydrolase family GH148. Sci Rep 7(1):17306 29229913 10.1038/s41598-017-16839-8 PMC5725463

[35] Nemmaru B, Ramirez N, Farino CJ, Yarbrough JM, Kravchenko N, Chundawat SPS (2021) Reduced type-A carbohydrate-binding module interactions to cellulose I leads to improved endocellulase activity. Biotechnol Bioeng 118(3):1141–1151 33245142 10.1002/bit.27637

[36] Courtade G, Forsberg Z, Heggset EB, Eijsink VGH, Aachmann FL (2018) The carbohydrate-binding module and linker of a modular lytic polysaccharide monooxygenase promote localized cellulose oxidation. J Biol Chem 293(34):13006–13015 29967065 10.1074/jbc.RA118.004269 PMC6109919

[37] Bolam DN, Ciruela A, McQueen-Mason S, Simpson P, Williamson MP, Rixon JE et al (1998) Pseudomonas cellulose-binding domains mediate their effects by increasing enzyme substrate proximity. Biochem J 331(Pt 3):775–781 9560304 10.1042/bj3310775 PMC1219417

[38] Hägglund P, Eriksson T, Collén A, Nerinckx W, Claeyssens M, Stålbrand H (2003) A cellulose-binding module of the Trichoderma reesei β-mannanase Man5A increases the mannan-hydrolysis of complex substrates. J Biotechnol 101(1):37–48 12523968 10.1016/s0168-1656(02)00290-0

[39] Zhang X, Rogowski A, Zhao L, Hahn MG, Avci U, Knox JP et al (2014) Understanding how the complex molecular architecture of mannan-degrading hydrolases contributes to plant cell wall degradation. J Biol Chem 289(4):2002–2012 24297170 10.1074/jbc.M113.527770 PMC3900950

[40] White JBR, Silale A, Feasey M, Heunis T, Zhu Y, Zheng H et al (2023) Outer membrane utilisomes mediate glycan uptake in gut Bacteroidetes. Nature 618(7965):583–589 37286596 10.1038/s41586-023-06146-w PMC7618045

[41] Tuson HH, Foley MH, Koropatkin NM, Biteen JS (2018) The Starch Utilization System Assembles around Stationary Starch-Binding Proteins. Biophys J 115(2):242–250 29338841 10.1016/j.bpj.2017.12.015 PMC6051301

[42] Dhawan S, Kaur J (2007) Microbial mannanases: an overview of production and applications. Crit Rev Biotechnol 27(4):197–216 18085462 10.1080/07388550701775919

[43] Bakshani CR, Cuskin F, Lant NJ, Yau HCL, Willats WGT, Grant Burgess J (2023) Analysis of glycans in a Burnt-on/Baked-on (BoBo) model food soil using Microarray Polymer Profiling (MAPP) and immunofluorescence microscopy. Food Chem 410:135379 36621331 10.1016/j.foodchem.2022.135379

